# Human antibody signatures towards the *Chlamydia trachomatis* major outer membrane protein after natural infection and vaccination

**DOI:** 10.1016/j.ebiom.2024.105140

**Published:** 2024-05-13

**Authors:** Ida Rosenkrands, Anja W. Olsen, Sara Knudsen, Nida Dehari, Helene Bæk Juel, Hannah M. Cheeseman, Peter Andersen, Robin J. Shattock, Frank Follmann

**Affiliations:** a Center for Vaccine Research, Department of Infectious Disease Immunology, Statens Serum Institut, Copenhagen, Denmark; b Department of Infectious Disease, Imperial College London, London, United Kingdom; c Novo Nordisk Fonden, Hellerup, Denmark

**Keywords:** *Chlamydia trachomatis*, Antibody, Neutralisation, Major outer membrane protein, Variable domain 4 (VD4), Infection, Vaccine

## Abstract

**Background:**

*Chlamydia trachomatis* (CT) Major Outer Membrane Protein (MOMP) holds a neutralising epitope in the Variable Domain 4 (VD4), and this region’s immune dominance during infection is well known. This study aimed to assess the antibody response induced after infection and compare it for specificity and functionality to the response following vaccination with the vaccine CTH522, which contains VD4’s from serovars D, E, F, and G.

**Methods:**

We assessed the antibody epitopes in MOMP by a high density peptide array. Furthermore, the role of the VD4 epitope in neutralisation was explored by competitive inhibition experiments with a fusion protein holding the neutralising VD4 linear epitope. This was done in two independent groups: 1) MOMP seropositive individuals infected with CT (n = 10, from case–control study) and 2) CTH522/CAF®01-vaccinated females (n = 14) from the CHLM-01 clinical trial.

**Findings:**

We identified the major antigenic regions in MOMP as VD4 and the conserved region just before VD3 in individuals infected with CT. The same regions, with the addition of VD1, were identified in vaccine recipients. Overall, the VD4 peptide responses were uniform in vaccinated individuals and led to inhibition of infection *in vitro* in all tested samples, whereas the VD4 responses were more heterogenous in individuals infected with CT, and only 2 out of 10 samples had VD4-mediated neutralising antibody responses.

**Interpretation:**

These data provide insights into the role of antibodies against MOMP VD4 induced after infection and vaccination, and show that their functionality differs. The induction of functional VD4-specific antibodies in vaccine recipients mimics previous results from animal models.

**Funding:**

This work was supported by the 10.13039/501100000780European Commission through contract FP7-HEALTH-2011.1.4-4-280873 (ADITEC) and 10.13039/501100006197Fonden til Lægevidenskabens Fremme.

**Research in context**

**Evidence before this study**

The *Chlamydia trachomatis* major outer membrane protein (MOMP) is an adhesin protein and the main antibody target during genital infection in humans. Antibodies against the MOMP and especially the variable domain 4 (VD4) can be neutralising, which has led to the inclusion of the VD4 into the vaccine CTH522.

**Added value of this study**

In individuals infected with *C. trachomatis*, the VD4 antibody responses were heterogenous and only a small proportion could neutralise infection *in vitro*. In contrast, the CTH522 vaccine-induced VD4 antibodies could neutralise infection *in vitro*, in agreement with previous results from animal models. These data show a difference in functionality of the infection- and vaccine-induced VD4 antibodies.

**Implications of all the available evidence**

Our results emphasise that the *C. trachomatis* infection drives a heterogenous antibody response against the VD4, in contrast to what is achieved after CTH522 vaccination. CTH522 vaccine-induced VD4 antibodies play a role in controlling chlamydial infections in animal models, in synergy with T cell responses. Further clinical studies may help to elucidate their specific function in the protective immune response in humans.

## Introduction

*Chlamydia trachomatis* (CT) causes sexually transmitted urogenital infections and is the most frequent cause of sexually transmitted bacterial disease in developed countries. Untreated infections in females can lead to sequelae in the upper genital tract, including pelvic inflammatory disease, ectopic pregnancy, and infertility. National control programs based on screening and treatment have not had significant impact on the incidence of urogenital CT infection. Therefore, an effective preventive vaccine has been defined as of high priority by the WHO.^1^

Studies of protective immunity to CT after urogenital infection in humans show that genital infections are cleared over time, the organism load is lower in individuals with repeated infections,^2^ and that there is a lower prevalence of CT reinfection early (<6 months) after the index visit,^3^ suggesting that a partial and short-lived protection is acquired by infection (reviewed by^4^). Also, reinfection is 4 times less frequent in women who spontaneously resolve infection.^5^ In a cohort of female sex workers from Nairobi, it was observed that the majority of widely spaced (>6 months) reinfections with CT were of different genotype, indicating that the immunity acquired after infection could be strain-specific.^6^ CT-specific CD4 IFN-γ responses have been associated with protective immunity against CT reinfection in humans,^7^ whereas infection-induced antibodies correlated with reduced cervical bacterial load.^8^

CT-specific antibodies are induced after genital infection in humans, and several CT molecules have been identified as targets, e.g., MOMP, OmcB, lipopolysaccharide, Hsp60 and Pgp3. However, the role of these antibodies in protection against reinfection in humans is not yet resolved. One possible protective mechanism would be bacterial neutralisation, however the neutralising activity of antibodies acquired after infection in humans has only been reported in a few studies. Sera from trachoma (ocular CT infection) patients demonstrated neutralising activity that was serovar (Sv)-specific (either SvA or SvB),^9^ and sera from individuals infected with SvD strains could neutralise SvD infection.^10^ Jones et al. reported that 65% of 34 women infected with CT had neutralising activity against the infecting strain,^11^ and Gupta et al. reported neutralising activity against SvD in women infected with different genotypes in a small study group (n = 19).^12^ Recently, Ardizzone et al. determined neutralisation titres in serum samples from 57 women diagnosed with genital CT infection of different genotypes and observed strong correlation between serum anti-elementary body (EB) IgG titres and neutralisation titres for SvD.^13^

CT MOMP is a glycosylated adhesin and the main target for antibodies induced during infection. MOMP has four surface-exposed variable domains (VD1 to VD4), and the classification of CT strains was originally based on antibody typing of MOMP (SvA-L). Infection-driven monoclonal antibodies directed at MOMP linear epitopes in VD1 (VAGLEK, VAGLQNDPT) and VD4 (LNPTIAG epitope) can be neutralising,^14^, ^15^, ^16^ and therefore MOMP has attracted much interest as a potential vaccine target, although the sequence variation between genotypes poses a potential challenge. The vaccine candidate CTH522 (an engineered construct based on MOMP that includes VD4 regions from SvD, E, F, and G) was designed to induce neutralising antibodies towards VD4, and this has been demonstrated in mouse and non-human primate models as well as in humans.^17^, ^18^, ^19^

Synthetic peptides from the VD4 region of MOMP are used in commercial ELISA tests,^20^ and approximately two thirds of women diagnosed with CT infection were seropositive in an IgG ELISA based on the VD4 peptide.^21^ In the light of this region being immune dominant during infection and a key component in the CTH522 vaccine, we found it intriguing to investigate the antibody responses it induces after natural infection and compare it with antibodies generated by CTH522 vaccination from a clinical phase I trial for specificity and functional capacity to neutralise CT infection.

In this study, we characterised the systemic antibody response in both naturally infected individuals and CTH522/CAF®01-vaccine recipients from a phase I study. We set out to study the antibody responses towards the linear immunodominant epitopes in VD4 in detail by performing epitope mapping. Furthermore, we determined CT surface recognition, *in vitro* neutralising capacity, and importantly, competitive neutralisation inhibition assays to reveal the role of VD4-specific antibodies in bacterial surface recognition and *in vitro* neutralisation. Here, we demonstrate that there is key difference in VD4-specific antibodies induced after infection and vaccination. A heterogenous VD4 epitope response is induced after CT infection, with only 2 out of 10 samples having VD4-mediated neutralising antibody responses, whereas a more uniform VD4 epitope response is observed after vaccination, with a clear association of neutralisation with the VD4 titre.

## Methods

### Study populations

The study included 80 individuals infected with CT (44 females and 36 males according to sex at birth) attending the sexually transmitted infections clinic at Bispebjerg Hospital, Copenhagen, who had tested positive for CT in a specific polymerase chain reaction (PCR) assay in the period June 17, 2003 till September 27, 2005.^22^^,^^23^ The median age was 23 (17–38 years) for females and 28 (20–66 years) for males. Three to four days after the CT test was performed a blood sample was drawn. The *ompA*-based genotype of the *C. trachomatis* isolates in urine was determined by direct sequencing of the full-length *ompA* gene after PCR amplification by the method of Stothard et al.^24^ Information on previous infections, and symptoms before diagnosis were included in the questionnaire. Blood donors with no history of positive CT testing were enrolled as control subjects (n = 17; 11 female and 6 male), the median age was 30 (21–60 years).

Furthermore, individuals from the CHLM-01 vaccine trial (NCT02787109) were part of this study. Females (according to sex at birth) were vaccinated with three intramuscular injections of CTH522/CAF®01-adjuvanted vaccine or placebo (saline) in the period August 15, 2016 till February 13, 2017. The median age was 24 (19–42 years) for CTH522/CAF®01-vaccinated (n = 14) and 23 (22–45 years) for placebo (n = 5). The enrolled participants had a negative urine PCR test for CT. The clinical trial has been reported by Abraham et al.^17^ Serum samples were collected 2 weeks after the third intramuscular immunisation for characterisation of the systemic antibody response after vaccination.

### Cultivation and harvesting of CT

CT SvD (UW-3/Cx, ATCC VR-885) elementary bodies (EBs) were prepared by infecting HeLa-229 cells (ATCC Cat# CCL-2.1, RRID:CVCL_1276) in RPMI 1640 medium (Gibco) containing 5% heat-inactivated foetal calf serum (FCS), 50 μg/ml gentamycin (Gibco) as previously described.^19^ Briefly, after harvesting the bacteria by two high-speed centrifugation steps, the suspended bacteria were sonicated, further purified on renografin cushion by ultracentrifugation, then resuspended in 250 mM sucrose, 10 mM NaH_2_PO_4_, 5 mM l-glutamic acid (SPG buffer) and stored at −80 °C. The inclusion-forming units (IFUs) of the batches were quantified by titration in HeLa-229 cells. For UV inactivation, EBs were treated by an UV lamp for 3–4 h on ice. Protein concentrations of UV-inactivated EBs (UV-EBs) were determined by the bicinchoninic acid protein assay (BCA assay, Thermo Fisher Scientific).

### Recombinant proteins

Recombinant proteins (MOMP or Ct681 and CT043 from SvD, VD4_6-22_^D/E/F/G^, and the negative control antigen MPT83 from *Mycobacterium tuberculosis*) were produced based on the amino acid sequences (from NCBI) with an added N-terminal six histidine tag. Synthetic DNA constructs were codon optimised for expression in *Escherichia coli*, followed by insertion into the pJexpress 411 vector (Atum). To avoid disulphide bridge formation during recombinant production, all cysteines in VD4_6-22_^D/E/F/G^ were exchanged with serines. Purification was done essentially as described elsewhere.^25^ Briefly, we induced expression in *E. coli* BL-21 (DE3) cells (Invitrogen) transformed with the synthetic DNA constructs by Isopropyl β-D-1-thiogalactopyranoside (IPTG). Inclusion bodies were isolated and extracts thereof were loaded on a HisTrap column (GE Healthcare), followed by anion exchange chromatography on a HiTrap Q HP column (GE Healthcare) and dialysis to a 20 mM glycine buffer, pH 9·2. Protein concentrations were determined by the BCA assay. CTH522 is a recombinant, engineered version of the CT MOMP and was produced in *E. coli* under Good Manufacturing Practice at Statens Serum Institut, as previously described.^26^

### Measurement of antibody levels by ELISA

Maxisorp Plates (Nunc, Denmark) were coated overnight with either recombinant proteins (1 μg/ml) or SvD UV-EBs (8 μg/ml) in carbonate buffer. Plates were washed with phosphate buffered saline (PBS) containing 0·2% Tween-20 and blocked with 2% skimmed-milk powder (SM) in PBS. The serum samples were titrated 2-fold in duplicates in PBS with 1% SM. Antigen-specific total IgG was detected with isotype-specific horseradish peroxidase (HRP)-conjugated Rabbit-anti-human (Agilent Cat# P0214, RRID:AB_2893418) and substrate TMB-PLUS (Kem-En-TEC, Denmark). Absorbance was recorded at 450 nm with subtraction of the absorbance value measured at 620 nm. For quantification of antibodies in samples, an in-house reference pool was established by combining samples from individuals with a positive response to SvD EBs. The pool was used to establish a standard curve for determination of titres in arbitrary ELISA Units/ml (AEU/ml) based on a five-parameter logistic curve using the package ‘drc’ in R. The cut-off in the MOMP and UV-EB ELISA was determined as mean + 3 standard deviations (SD) of the response in controls.

ELISA reactivity against the 9-mer overlapping biotinylated peptide PepSet library (overlap of 8 amino acids) spanning the VD4 region of SvD was investigated as previously described.^19^ Briefly, ELISA plates were coated with streptavidin or neutravidin, incubated with biotinylated peptides (Mimotopes, United Kingdom), blocked with 2% SM in PBS, washed, and then the normal ELISA procedure was followed. Absorbance values above 0·2 were considered positive.

In ELISA inhibition experiments, diluted samples were incubated in duplicates with antigen diluted in SPG buffer for 45 min at 37 °C before the standard ELISA.

### Western blot

Mini-PROTEAN® TGX™ 4–20% precast gels (BIO-RAD, Hercules, CA) were used, and after electrophoresis, CT SvD EB samples were transferred to nitrocellulose membranes. The membranes were blocked with 5% SM in PBS with 0·1% Tween-20, cut into strips representing each lane followed by incubation of the strips with serum samples diluted in 1% SM in PBS with 0·1% Tween-20, and detection by HRP-conjugated rabbit-anti-human secondary antibodies (Agilent Cat# P0214, RRID:AB_2893418) for chemiluminescence with ECL Plus reagent (GE Healthcare) on a G:BOX Chemi XX6 instrument.

### Peptide array analysis

The array consisted of triplicates of immobilised 15-mer peptides overlapping by 14 amino acids. The peptides were synthesised based on sequences of CT MOMP proteins from SvD/UW-3/CX (Genbank ID AAC68276.1), SvE Bour (CAA36791.1), SvF IC-CAL3 (CAA36299.1), and SvG G/UW-57/Cx (BAO53911.1), and were printed on functionalised glass slides, attached through a linker on the N-terminus of the peptides by JPT Peptide Technologies using PepStar™ technology. Serum samples from controls, infected subjects or CTH522/CAF®01-vaccinated individuals were diluted 1:200 in Superblock TBS T20 buffer (Thermo Fisher) and incubated at 30 °C for 2 h, washed, and IgG peptide complexes were visualised using Alexa Fluor 647-labelled secondary anti–human IgG. After washing and drying, slides were scanned with a high-resolution scanner at 635 nm and data reported as arbitrary fluorescence units. We used the 10% of maximum intensity signal as cut-off for a positive response. The mean of triplicate values was determined; however, if the coefficient of variation was larger than 0·5, only the 2 closest values were used.

### *In vitro* neutralisation assay

The assay was performed essentially as described elsewhere.^27^^,^^28^ Briefly, Syrian golden hamster kidney (HaK) cells (ATCC Cat# CCL-15, RRID:CVCL_3315) were seeded in 96-well flat-bottom microtiter plates. Samples were heat-inactivated at 56 °C for 30 min and diluted in SPG buffer to create 2-fold dilution series in duplicates starting from 10-fold dilution. The CT stocks were mixed 1:1 with the diluted samples and incubated for 45 min at 37 °C. The mixture was used for infection of HaK cells for 2 h at 35 °C, and thereafter the cells were incubated for 24 h. Chlamydial inclusions were visualised by staining with polyclonal rabbit anti-recombinant CT043 serum, followed by Alexa Fluor 488-conjugated goat anti-rabbit immunoglobulin (Thermo Fisher Scientific Cat #A11008, RRID:AB_143165). Cells were stained with propidium iodide (Invitrogen) and IFUs were counted by an ImageXpress Pico automated cell imaging system (Molecular Devices, San Jose, CA) as previously described.^28^ A positive and negative reference pool were established by combining samples from individuals with a positive neutralisation response and controls, respectively, and the reference pools were included on all plates. Percent specific neutralisation was calculated as 100 × (No sample control IFU – sample IFU)/No sample control IFU. The serum dilution giving a 50% reduction in IFU was named reciprocal 50% neutralisation titre (NT_50_). NT_50_ values were calculated based on a 5-parameter logistic curve using the package ‘drc’ in R. For samples where no titre could be calculated, the samples were assigned a titre of 5 (half the value of the lowest dilution). The threshold titre for positivity was ≥50 (five times the minimum detection limit of 10).

In neutralisation-inhibition experiments, diluted samples were incubated with antigen diluted to 160 μg/ml in SPG buffer for 45 min at 37 °C before the incubation step with bacteria. Results are presented as specific neutralisation calculated as described above or as relative infection (%) that is 100 × IFU (sample plus antigen) ⁄ IFU (no sample, no antigen).

### Reagent validation

The cell lines were validated by the vendor (ATCC), this included also STR profiling for the HeLa-229 cell line. At receipt, cell lines were propagated, aliquoted and freezed according to the instructions by ATCC and mycoplasma test was performed.

Commercial antibodies were validated by the vendor. The CT043 rabbit antibody was generated by immunising rabbits with recombinant CT043. The antibody was validated for specificity and reactivity by testing serum samples for CT043 response before and after immunisation by ELISA and for reactivity towards CT infected and non-infected HaK cells by fluorescence microscopy.

### Ethics

The studies involving human participants infected with CT were reviewed and approved by the Local Ethics Committee for Copenhagen (reference number 01-008/03), all participants gave written informed consent before enrolment, The CHLM-01 study was done in accordance with the International Conference on Harmonization’s Good Clinical Practices guidelines.^29^ All participants gave written informed consent before enrolment, and the study protocol was reviewed and approved by the London–Chelsea Research Ethics Committee, the Research and Development department at Imperial College Healthcare National Health Service (NHS) Trust, and the Medicines and Healthcare Products Regulatory Agency (EudraCT number 2015-004330-10).

### Statistics

R (version 4·0·0) and GraphPad Prism (9·0·2) (RRID:SCR_002798) software was used for data handling, analysis, and graphic representation. Antibody and neutralisation titres are presented as median and interquartile range, and compared by the Kruskal–Wallis test with Dunn’s post-test. Correlation analysis was performed with non-parametric Spearman’s rank correlation coefficient. In neutralisation inhibition experiments, comparisons of preincubations with different antigens were analysed by one-way ANOVA with Dunnett’s correction for multiple comparisons. In all analyses, a p value < 0.05 was considered significant. ∗p < 0.05, ∗∗p < 0.01, ∗∗∗p < 0.001, ∗∗∗∗p < 0.0001.

### Role of funders

The funders had no role in study design, data collection and analysis, decision to publish, or preparation of the manuscript.

## Results

### Description of study participants

In the CHLM-01 clinical trial 35 females were randomly assigned to receive CTH522/CAF®01 (n = 15), CTH522 adjuvanted with aluminium hydroxide, CTH522/AH (n = 15), or placebo (n = 5), and serum anti-CTH522 IgG responses, CT surface recognition, and neutralisation titres have been previously published.^17^^,^^30^ CTH522/CAF®01 induced higher antibody titres and cell-mediated immune responses than CTH522/AH, and CTH522/CAF®01 was therefore selected for a more detailed characterisation in the present study. Fourteen participants (V1–V14) in the CTH522/CAF®01 group completed all three intramuscular immunisations.

From a cohort of individuals genitally infected with CT, 80 participants were included in this study^22^^,^^23^ (Supplemental Table S1). DNA sequencing of the isolated CT strains demonstrated that genotype E was most frequent (34%) followed by D (24%) and F (20%). Previous infection was self-reported for 29% of the individuals infected with CT. Among males, 56% reported symptoms before diagnosis, whereas only 32% of the females observed symptoms prior to diagnosis.

### Characterisation of infection-driven antibody responses

**Fig. 1:**
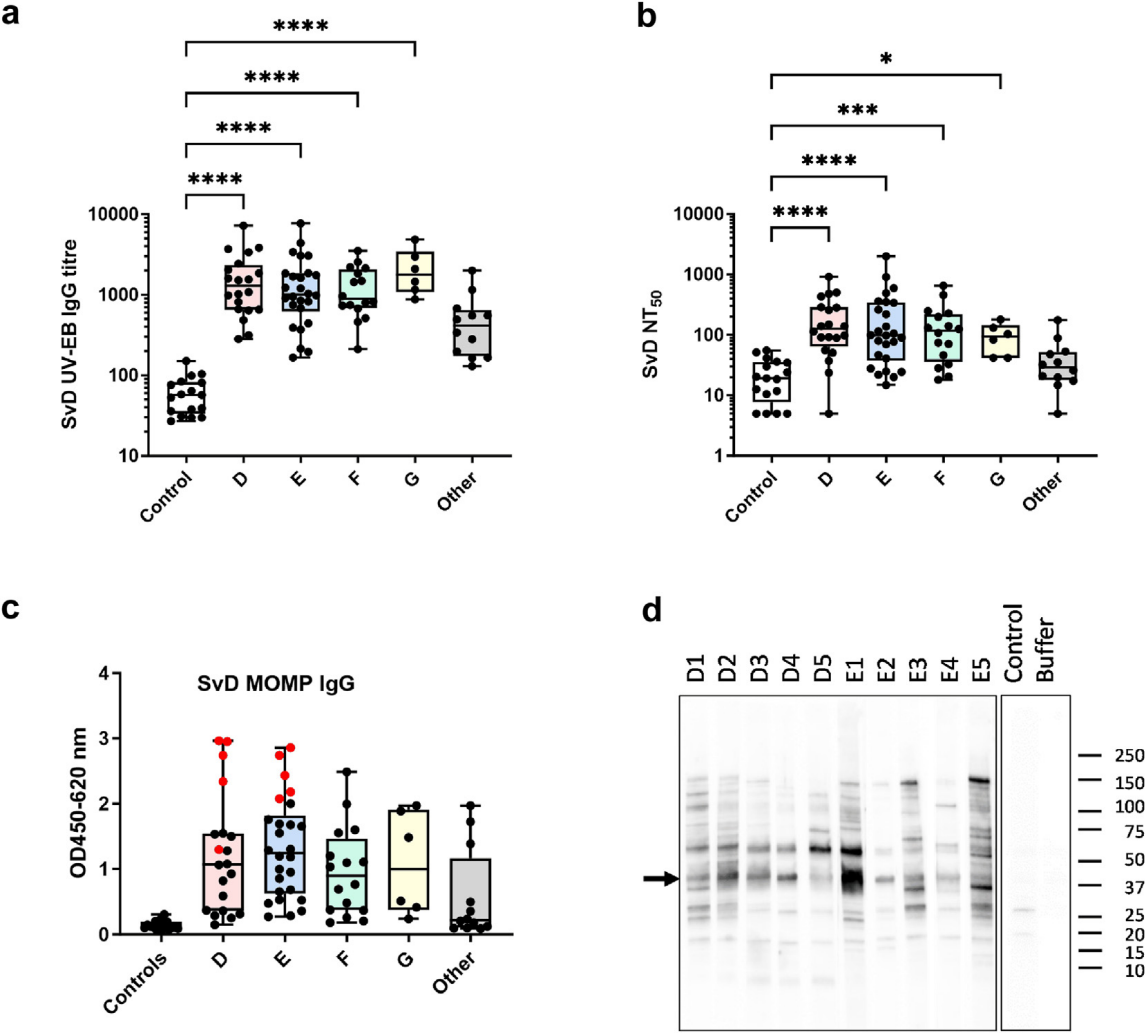
The infection driven antibody response. **a)** The IgG response to SvD UV-EBs in individuals infected with CT and grouped according to the infecting strain and controls. An in-house reference serum pool and the samples were diluted 1:100 (controls 1:25) and further in 2-fold serial dilutions. The concentration of anti-UV-EB IgG antibodies was calculated as arbitrary ELISA units using the reference serum pool as the calibrator. **b)** Neutralisation of SvD infection of HaK cells was determined in individuals infected with CT and controls. The reciprocal serum dilution giving a 50% reduction in IFU, NT_50_, was determined. The results are presented according to the infecting strain as median and interquartile range. Infected and controls were compared by the Kruskal–Wallis test with Dunn’s post test. **c)** Ten samples from individuals infected with CT with a high ELISA response to SvD MOMP (red points) were selected and named according to the genotype of the infecting strain (D1–D5 and E1–E5). **d)** Western blot IgG analysis with SvD EBs for D1–D5, E1–E5 and negative control. The position of MOMP is indicated by an arrow and the position of molecular mass marker bands are shown.

With the overall aim to describe and compare the antibody response in CT-infected and CTH522-vaccinated individuals, we started out by studying CT surface recognition and neutralising capacity of sera from the cohort of individuals infected with CT. The CT B-complex D and E strains accounted for 58% of the genotyped strains, and the strains are genetically closely related. We selected SvD as the strain representing this complex and measured IgG responses towards SvD UV inactivated EBs (UV-EB) in blood samples. For SvD UV-EBs, the median titre was 984 (Fig. 1a), and 79 out of 80 (99%) had positive responses. The control group of donors with no history of CT infection had median UV-EB titres of 57, and none of them had positive responses. Compared to the controls, all groups except other genotypes (SvH-K) showed significantly higher median titres (p < 0·0001, Kruskal–Wallis). No significant difference was found between responses in males (median UV-EB titre of 913) and females (median UV-EB titre of 1117) (p = 0·3646, Kruskal–Wallis).

Next, we evaluated the ability of antibodies to inhibit CT infection *in vitro* by determining the 50% reciprocal neutralising titres, NT_50_, against CT SvD for the individuals infected with CT and controls. The analysis was performed using the standard protocol previously developed for the Chlamydia community^27^ using Syrian hamster kidney (HaK) cells, which contain no Fc receptors that could interfere with the assay.^31^ SvD NT_50_ values in individuals infected with CT had a median titre of 92; for controls, the median titre was 19 (Fig. 1b). SvD NT_50_ values above 50 were found in 66% of individuals infected with CT and 12% of controls. Although not statistically significant, the median NT_50_ values were higher in females (104) than males (79) (p = 0·1733, Kruskal–Wallis). A moderate positive correlation between the EB titre and NT_50_ was observed (r = 0·576, p < 0·0001, Spearman).

Since MOMP is immunodominant during infection and the target for further investigations, we analysed the SvD MOMP antibody response of individuals infected with CT in order to select samples for a more detailed characterisation. Overall, 63 out of 80 (79%) had a positive response to SvD MOMP. We selected ten samples with high ELISA responses to MOMP (OD > 1·0, Fig. 1c) to ensure a detectable response by a peptide microarray assay, which in general, has a lower sensitivity than ELISA. Five of the selected individuals were infected with genotype D (D1–D5), and five were infected with genotype E (E1–E5). Western blot analysis with SvD EBs confirmed the recognition of the band representing MOMP for these ten donors (marked by arrow, Fig. 1d), who were thereafter named ‘infected MOMP-high-responders’. Among these individuals, 7 (70%) were females, and 5 (50%) had reported a previous CT infection.

### MOMP epitope mapping of sera from CT-infected and CTH522-vaccinated individuals

**Fig. 2:**
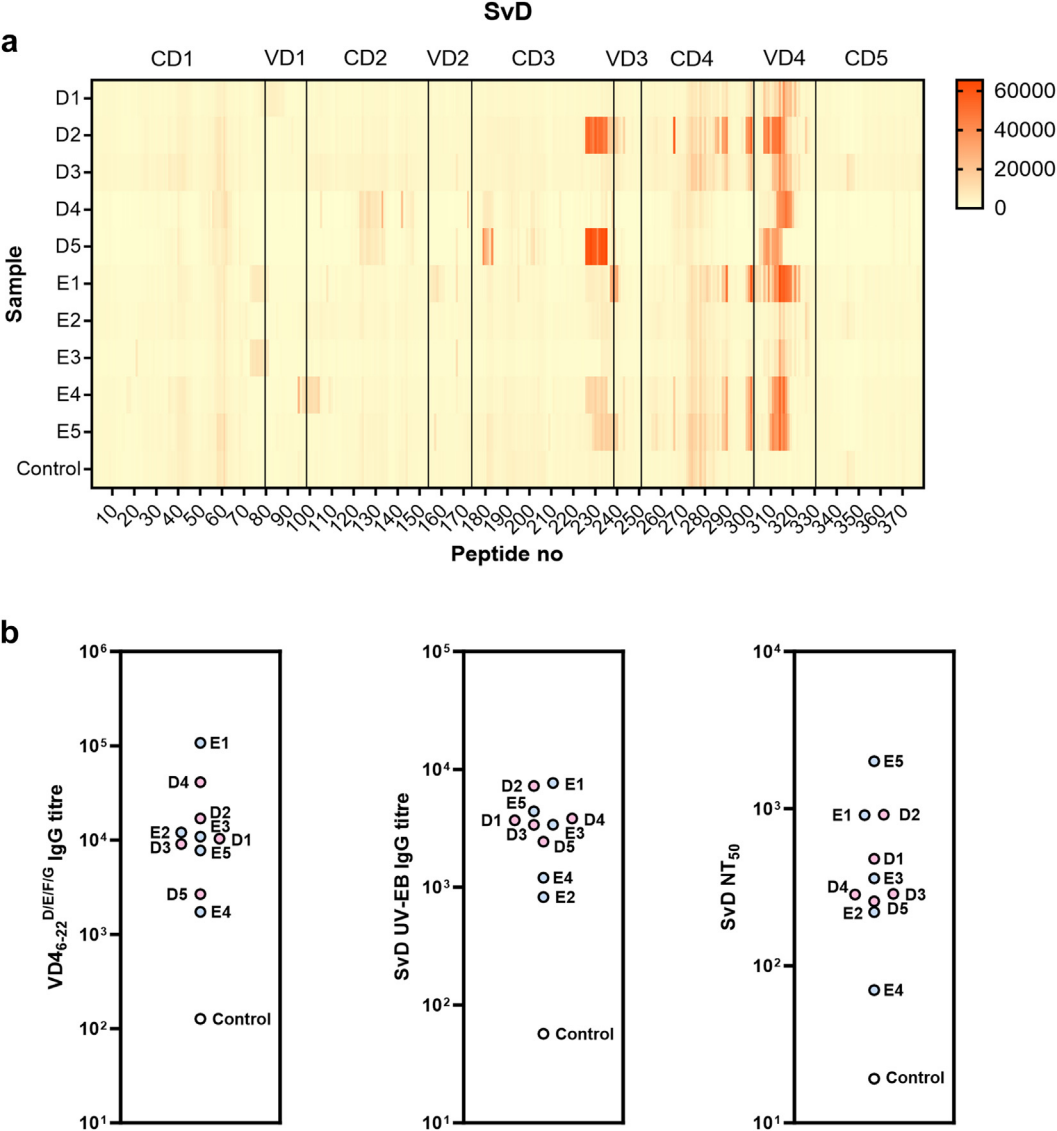
Epitope mapping of MOMP in individuals infected with CT. **a)** Heat map of IgG responses to MOMP SvD 15-mer overlapping peptides in peptide array for infected MOMP-high-responders D1–D5, E1–E5 and negative control. Responses are shown as mean fluorescence intensity for each peptide. Constant domains (CD) and variable domains (VD) in MOMP are indicated. **b)** IgG ELISA titres measured for VD4_6-22_^D/E/F/G^ and SvD UV-EBs, and SvD NT_50_ values are shown for D1–D5, E1–E5 and negative control. Determination of the IgG titres was calculated as arbitrary ELISA units using a reference serum pool as the calibrator.

Peptide microarray-based characterisation of antibody responses to MOMP in the ten infected MOMP-high-responder samples was then performed with 15-mer peptides with an overlap of 14, covering the entire sequence of MOMP from SvD (Fig. 2a, Supplemental Table S2). We detected epitopes throughout the molecule, but two regions were recognised more frequently: Response to the Constant Domain 3 (CD3)/VD3 region was observed in five out of ten samples, and response to the VD4 region was observed in all ten samples. In the VD4 region, all samples responded to peptides containing the sequence DTTTLNPTIAGAGD. The same analysis was performed for the MOMP SvE sequence (Supplemental Fig. S1 and Table S2), and an overview of the location of antigenic regions in the MOMP SvD and SvE amino acid sequences is shown in Supplemental Fig. S2. The result for SvE is highly similar to that of SvD, however, we observed that an antigenic region (VELYTDTTFAW) found in CD3 was specific for SvD, whereas a region (STTGN) found in VD1 was specific for SvE.

VD4_6-22_^D/E/F/G^ is a recombinant protein designed to contain 17-amino acid-stretches holding the known neutralising linear epitope LNPTIAG from each of the Sv D (FDTTTLNPTIAGAGDVK), E (FDTTTLNPTIAGAGDVK), F (VDITTLNPTIAGSGSVA), and G (VDITTLNPTIAGSGSVV) designated VD4_6-22_^D/E/F/G^. These regions are also included in the CTH522 vaccine construct. To further zoom in and quantify the antibody response specifically towards the VD4 region, we measured antibody responses against VD4_6-22_^D/E/F/G^ by ELISA. Titres for VD4_6-22_^D/E/F/G^ and SvD UV-EBs determined by ELISA and the SvD NT_50_ values are shown for all ten infected MOMP-high-responders (Fig. 2b). Median SvD UV-EB titres (3554) and SvD NT_50_ values (324) in theses donors were 3·5 and 3·6 times higher, respectively, compared to the overall panel of individuals infected with CT. For the infected MOMP-high-responders, no significant correlation between VD4_6-22_^D/E/F/G^ titres and SvD NT_50_ values was observed (r = 0·3939, p = 0·2632, Spearman).

**Fig. 3:**
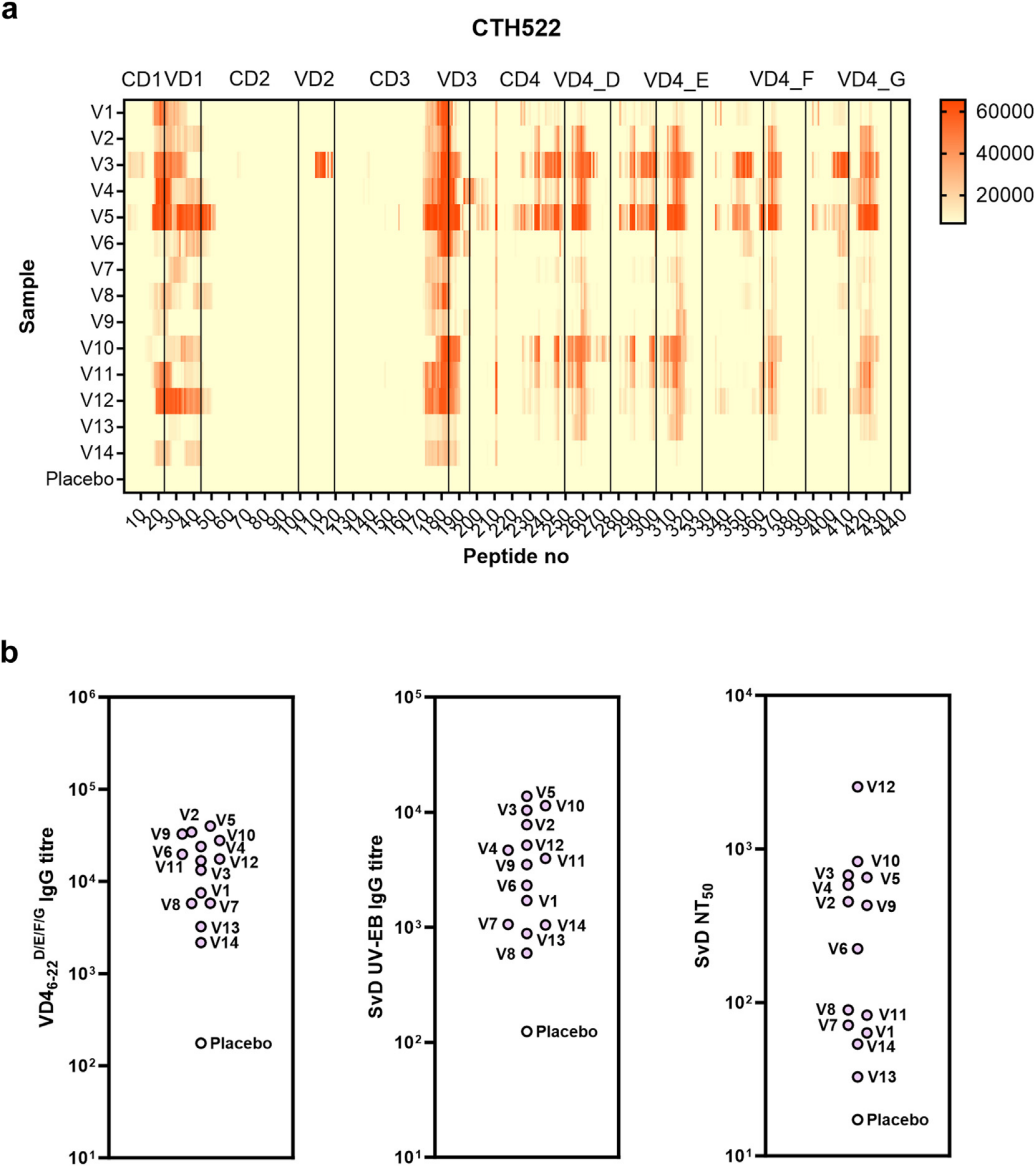
Epitope mapping of CTH522 in vaccinated individuals. Fourteen participants were included from the CHLM-01 trial after 3 intramuscular immunisations with CTH522/CAF®01. **a)** Heat map of IgG responses to CTH522 15-mer overlapping peptides in peptide array for participants vaccinated with CTH522/CAF®01 and placebo negative control. **b)** IgG ELISA titres for VD4_6-22_^D/E/F/G^ and SvD UV-EBs, and SvD NT_50_ values determined in participants vaccinated with CTH522/CAF®01 and placebo negative control.

To compare the responses in infected and vaccinated individuals, we performed similar analyses for the CTH522/CAF®01-vaccinated CHLM-01 participants. CTH522 has SvD MOMP as backbone (amino acids 34–259) followed by extended VD4 regions from SvD (amino acids 260–327), SvE, SvF, and SvG, which all contain the neutralising epitope.^19^ The peptide array analysis demonstrated a strong recognition of the VD1, CD3/VD3 and CD4/VD4 regions by all CTH522/CAF®01 participants, and one participant strongly recognised the VD2 region (Fig. 3a, Supplemental Table S2). An overview of the antigenic regions in CTH522 is shown in Supplemental Fig. S3. The consistent strong response to CD1/VD1 and CD3/VD3 was a notable difference compared to the response in individuals infected with CT (Fig. 2a). In the VD4 region, all samples responded to peptides containing the sequence TTTLNPTIAGAGD. ELISA titres for VD4_6-22_^D/E/F/G^ and SvD UV-EBs and SvD NT_50_ values are shown also for the vaccine recipients (Fig. 3b). We noted that the determined range of titres for both VD4_6-22_^D/E/F/G^, UV-EBs, and NT_50_ are at the same level in the infected MOMP-high-responders and the CTH522/CAF®01-vaccinated participants (Figs. 2b and 3b). Importantly, after vaccination a strong positive correlation between VD4_6-22_^D/E/F/G^ and SvD NT_50_ values was observed (r = 0·6923, p = 0·0077, Spearman).

**Fig. 4:**
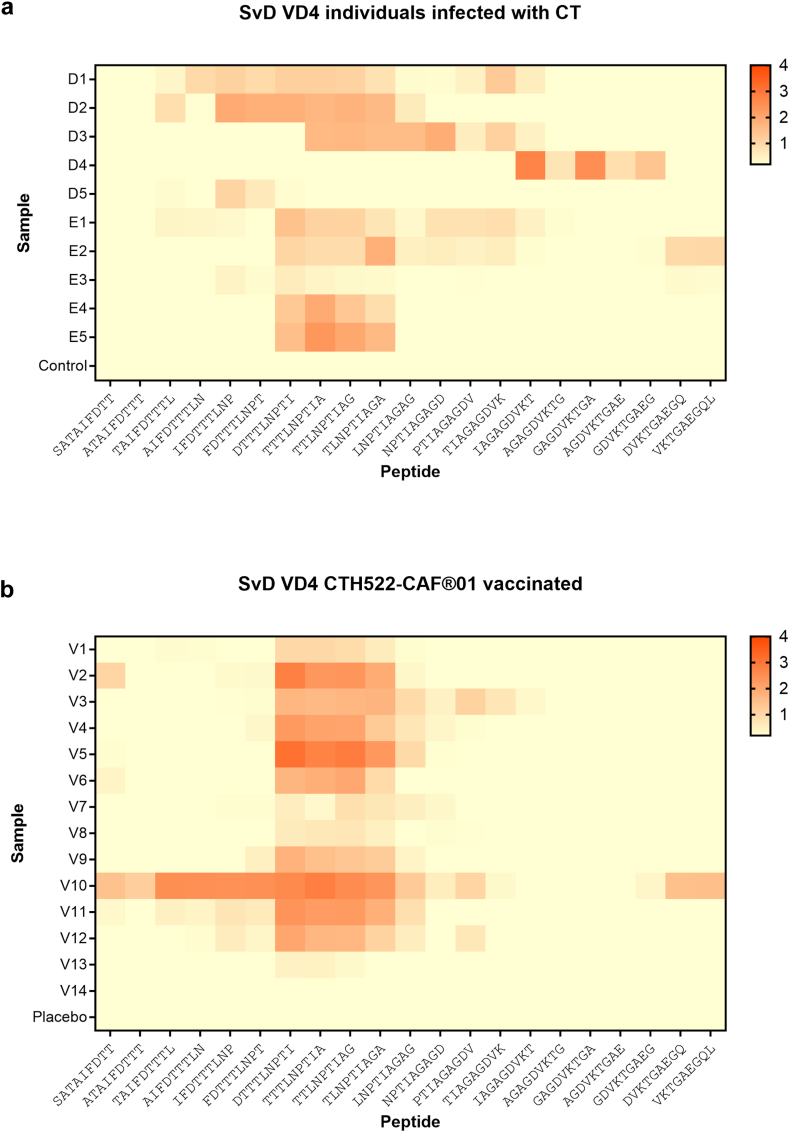
Antibody response to MOMP VD4. A heat map of IgG responses to SvD MOMP VD4 overlapping 9-mer peptides as detected by ELISA (represented as OD450-620 nm values) is shown for **a)** the infected MOMP-high-responders (D1–D5, E1–E5) and negative control and for **b)** CTH522/CAF®01-vaccinated individuals and placebo negative control.

The IgG response to SvD VD4 was further examined by overlapping 9-mer peptides covering the VD4 sequence SATAIFDTTTLNPTIAGAGDVKTGAEGQL (Fig. 4a). Some heterogeneity in the response pattern was observed across the ten selected samples from individuals infected with CT, but most samples (8 out of 10) responded to peptides containing the TLNPTI sequence. However, for samples D4 and D5, the 9-mer responses were skewed either C-terminally or N-terminally, respectively. Overall, the 9-mer responses were located in the same region of VD4 as observed for the 15-mer responses and served to narrow down the amino acid sequence of the epitope(s) recognised in VD4 for most samples. However, for D4, the 9-mer responses were skewed to the right compared to the 15-mer responses (Supplemental Table S2), which could reflect that more than one VD4 epitope is present in this sample, and that the other epitope is longer than 9 amino acids and therefore not presented by the 9-mer PepSet.

For the vaccinated individuals, a more uniform response was observed to the overlapping 9-mer peptides covering VD4 from SvD (Fig. 4b). All samples had a positive response to peptides containing the TLNPTI sequence. However, in particular one participant (V10) had a very broad response to the 9-mers.

### Blocking of bacterial surface recognition

Neutralisation of CT infectivity requires that antigenic regions are surface-exposed and accessible for antibody binding. Therefore, as preparation for neutralisation-blocking experiments, we tested the contribution of infection- and vaccine-induced VD4 antibodies in surface recognition by competitive inhibition ELISA. First, we evaluated if the VD4_6-22_^D/E/F/G^ protein could block antibody binding in a VD4_6-22_^D/E/F/G^ ELISA and SvD UV-EB ELISA.

Samples from the ten infected MOMP-high-responders all had positive responses to VD4_6-22_^D/E/F/G^ in ELISA (Fig. 2b) and recognised the peptides containing DTTTLNPTIAGAGD including the neutralising epitope LNPTIAG (Fig. 4a). We started by evaluating the blocking effect of VD4_6-22_^D/E/F/G^ and a control antigen (a secreted protein from *M. tuberculosis*) in a VD4_6-22_^D/E/F/G^ ELISA. Samples were diluted corresponding to approximately 80–90% neutralisation and pre-incubated with VD4_6-22_^D/E/F/G^ or the control protein before the ELISA (Supplemental Fig. S4a). For all samples, the ELISA response to VD4_6-22_^D/E/F/G^ was effectively blocked by adding VD4_6-22_^D/E/F/G^ in a concentration of 160 μg/ml. The same blocking experiments were performed with samples from vaccinated individuals, selecting the seven participants with SvD NT_50_ titres above 200. Likewise, samples were diluted corresponding to approximately 80–90% neutralisation and competitive inhibition was done with different proteins in a fixed concentration of 160 μg/ml. With these samples we also observed that the ELISA response to VD4_6-22_^D/E/F/G^ was completely blocked by adding VD4_6-22_^D/E/F/G^ in this concentration (Supplemental Fig. S4b).

**Fig. 5:**
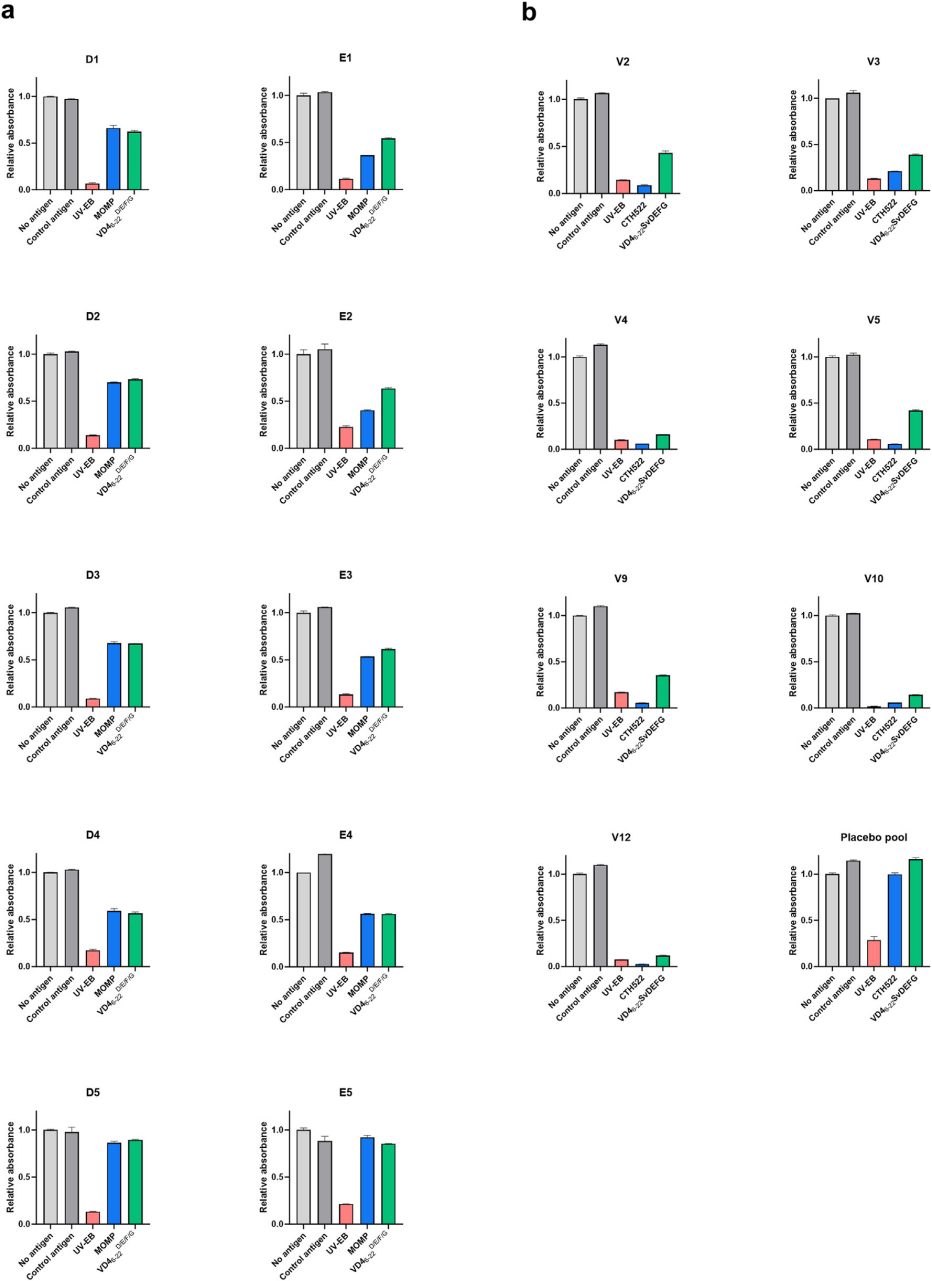
Blocking of ELISA response with different CT antigens. ELISA plates were coated with SvD UV-EB and samples from **a)** infected or **b)** vaccinated individuals were diluted corresponding to 80–90% neutralisation against SvD and pre-incubated with 160 μg/ml of control antigen, VD4_6-22_^D/E/F/G^, CTH522 or SvD MOMP, SvD UV-EBs or no antigen before the standard ELISA. For each antigen, absorbance relative to that of the no antigen is shown. Results are presented as mean plus standard deviation.

In the same manner, we tested the blocking effect of SvD UV-EB, SvD MOMP and VD4_6-22_^D/E/F/G^ in a SvD UV-EB ELISA with samples from the infected MOMP-high-responders (Fig. 5a). The surface binding was blocked by adding UV-EB in a concentration of 160 μg/ml for all samples. The blocking with MOMP and VD4_6-22_^D/E/F/G^ was identical for most samples, however for E1, E2, and E3, a higher blocking effect was obtained with MOMP, reflecting that MOMP epitopes outside VD4 are presented on the surface for these samples. These three samples also showed recognition of VD1 in the peptide array, so it is possible that a linear VD1 epitope is also present on the surface of the SvD EBs. For D5, no blocking was observed with MOMP and VD4_6-22_^D/E/F/G^ suggesting that the VD4 epitope recognised by D5 is not presented on the surface of the bacteria. With samples from vaccinated individuals, complete blocking was obtained with UV-EB and CTH522, whereas the blocking effect of VD4_6-22_^D/E/F/G^ was only partial for some participants, reflecting recognition of MOMP epitopes outside VD4 on the surface for these samples (Fig. 5b).

### Blocking of neutralisation

**Fig. 6:**
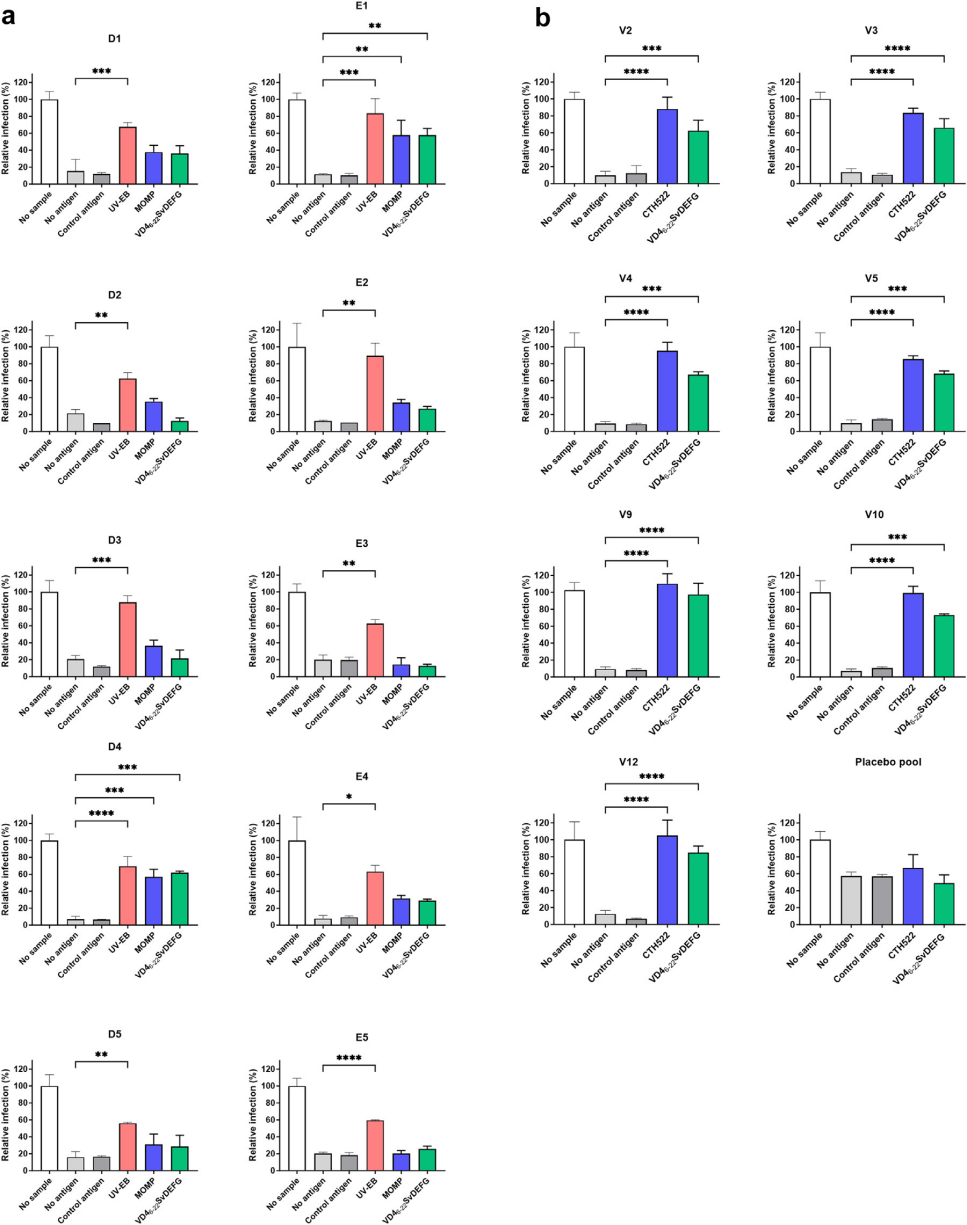
Neutralisation blocking assay with different CT antigens. Each sample was diluted to obtain 80–90% neutralisation and preincubated with 160 μg/ml of the antigens **a)** SvD UV-EBs, SvD MOMP, VD4_6-22_^D/E/F/G^, a control antigen or no antigen (individuals infected with CT) or 160 μg/ml of the antigens **b)** CTH522, VD4_6-22_^D/E/F/G^, a control antigen, or no antigen (vaccinated individuals) before the standard neutralisation assay. For each treatment, CT infection relative to that of the no sample group (only CT bacteria) is shown. Results are presented as mean plus standard deviation, and for each donor, comparisons of different antigen preincubations were analysed by one-way ANOVA.

Having shown the blocking effect of SvD UV-EB, SvD MOMP or CTH522, and VD4_6-22_^D/E/F/G^ on surface recognition, we continued to test the blocking effect on neutralisation for the infected MOMP-high-responders (Fig. 6a). The CT infection is presented as relative to the infection level when no sample is added in the assay. For each donor, comparison of the blocking effect of different antigens were analysed by one-way ANOVA. Serum samples were diluted corresponding to approximately 80–90% neutralisation. Blocking with the control antigen did not affect the neutralising activity, whereas blocking with SvD UV-EB resulted in significant loss of neutralisation and thereby increase in infection for all samples. For D4 and E1, MOMP and VD4_6-22_^D/E/F/G^ blocking also resulted in significant loss of neutralisation and increase in CT infection, demonstrating that VD4 serves as a neutralising antibody epitope for these samples. In all other samples, blocking with MOMP and VD4_6-22_^D/E/F/G^ did not lead to significant increase in infection.

As for the surface recognition, CTH522/CAF®01-vaccinated showed a much clearer correlation between antigen recognition and neutralising effect. In all samples, blocking with CTH522 or VD4_6-22_^D/E/F/G^ resulted in significant increase in relative infectivity (Fig. 6b), thereby confirming that antibodies against the neutralising epitope in VD4 are induced by vaccination.

## Discussion

We investigated the human antibody responses towards CT MOMP after infection and vaccination. Using detailed epitope mapping, determination of bacterial surface recognition and *in vitro* neutralising capacity, we confirm the immunodominant role of antibodies towards the VD4 region of MOMP during genital infection, in patients visiting an outpatient clinic. We show here that this immune dominance rarely leads to an effective neutralising response following infection, and that VD4 responses do not correlate with ability to neutralise. In contrast, vaccination with the vaccine candidate CTH522, which includes VD4 regions from SvD-G, induced neutralising antibodies in a recent phase I trial,^17^^,^^30^ and we show here that there is a clear association of neutralisation with the VD4 titre. Epitope mapping indicates that the more uniform VD4 epitope response, observed after CTH522/CAF®01 vaccination, is associated with neutralising capacity.

Previous studies have shown that MOMP is the main target of antibodies induced during a genital CT infection in humans.^32^^,^^33^ In line with this, we found frequent recognition of both SvD UV-EBs and recombinant SvD MOMP, with 99 and 79%, respectively, of all individuals infected with CT giving a positive antibody response. Individuals infected with genotypes D, E, F, and G showed significantly higher titres compared to the controls, whereas the titres in individuals infected with other genotypes (Sv H, I, J, and K) were lower (Fig. 1a). Differences in serological response to infections with the B-complex and intermediate B-related strains (D, E, F, G) compared to C-complex strains (A, C, H, I, and J) are likely linked to a differential surface exposure of the VD4 region, as previous studies by Zhong et al. reported that the VD4 TLNPTIAG epitope was present on the surface of SvA, D, E, F, K, L2, and L3, but not on the surface of the C-complex SvC, H, I, and J.^34^

We dissected the infection-driven antibody signatures towards MOMP in a subset of CT-infected (genotypes D and E) individuals with high responses to recombinant MOMP (Fig. 2). Results from the peptide array analysis demonstrated that VD4 is immunodominant (10 out of 10 samples) after genital infection in humans. This is in agreement with other studies using different approaches (e.g.,^35^) and also with a recent study, which reported 65·5% positive samples with a commercial MOMP VD4 peptide based ELISA in women tested within 3 months of notification of infection.^21^ Furthermore, the CD3/VD3 region was recognised in the peptide array by 5 out of 10 samples in this study as also observed by Collar et al.^35^

The CTH522 vaccine-induced serum responses strongly recognised the CD1/VD1, CD3/VD3 and CD4/VD4 regions in all CTH522/CAF®01 recipients by peptide array analysis (Fig. 3). The consistently strong response to CD1/VD1 and CD3/VD3 was a notable difference compared to the response in individuals infected with CT.

The protective effect of neutralising antibodies against CT infection has been shown in mice. Passive transfer of IgG/serum and *in vivo* neutralisation experiments have demonstrated the ability of antibodies to significantly protect against CT infection.^19^^,^^36^^,^^37^ Here, using the HaK cell line for CT *in vitro* neutralisation assay,^27^ we observed that SvD NT_50_ values above 50 were found in 66% of individuals infected with CT (Fig. 1b). To date, only a few studies have assessed the neutralising effect of serum samples after genital infection in larger cohorts. Ardizzone et al. determined the NT_50_ levels against SvD and SvIa in infected women using the A2EN cell line,^13^ and Gupta et al. using Hep2 cells.^12^ The HaK cell line used in this study has previously shown not to be affected by the antibody isotype and is therefore a good cell line for studying complement-independent, antibody-mediated neutralisation of chlamydial infectivity.^31^

Since the VD4 region is a well-known target for antibodies during infection, and in animal models can induce neutralising and protective antibodies, we studied to what extent the natural infection in humans drives a neutralising response against VD4, and compared that to the response generated by the recombinant vaccine CTH522, designed to induce neutralising antibodies against VD4. To study this, we used the VD4_6-22_^D/E/F/G^ construct that contains all the VD4 epitopes included in the CTH522 vaccine construct.^19^ Notably, the VD4_6-22_^D/E/F/G^, SvD UV-EB and NT_50_ titres are comparable between the infected MOMP-high-responders and the CTH522/CAF®01-vaccinated participants (Figs. 2b and 3b). Blocking experiments with the VD4_6-22_^D/E/F/G^ protein, showed that VD4 epitopes are responsible for the neutralising effect in two out of ten individuals infected with CT, whereas in the other individuals, VD4 contributed only marginally or not at all to the neutralising effect (Fig. 6a). In the ten individuals infected with CT, VD4_6-22_^D/E/F/G^ and recombinant MOMP provided the same level of inhibition, indicating that VD4 holds the major linear neutralising epitope in SvD MOMP. A strong positive correlation was observed between VD4 titre and the neutralising response for the CTH522/CAF®01 recipients, and CTH522 and VD4_6-22_^D/E/F/G^ both blocked the neutralising effect in all samples evaluated (Fig. 6). In the same manner, a strong positive correlation between SvD neutralisation and VD4 responses was observed in mice after vaccination with CTH522/CAF®01.^30^

The TTLNPTIAG sequence in VD4 is conserved across serovars, and this region has been suggested to form a partially immune-accessible cleft in the VD4 loop, which also serves as a site for attachment to the epithelial cell.^38^^,^^39^ All individuals infected with CT evaluated by peptide array showed recognition of the VD4 region, but the responses to the individual 9-mer peptides in the VD4 region showed variation between the donors (Fig. 4a). Although 8 out of 10 samples responded to peptides containing the TLNPTIA sequence, the 9-mer responses for samples D4 and D5 were skewed either to the right or left, respectively. This may reflect that multiple, overlapping B cell-epitopes are present in VD4 (reviewed by^40^), and that the antibody responses to this region could also originate from potential contiguous or discontinuous epitopes.^34^^,^^41^ These epitopes would depend on correct folding of the protein and are not expected in a recombinant form of MOMP like CTH522.^26^ In line with this, CTH522/CAF®01-vaccinated individuals showed a much more uniform response to the 9-mer and 15-mer peptides representing VD4, and all samples responded to 9-mer peptides containing the sequence TLNPTI (Fig. 4b). The presence of overlapping epitopes in VD4 makes a more detailed definition of individual VD4 epitopes in these polyclonal samples challenging. A shift by just one amino acid in the linear epitope may disrupt the surface recognition,^41^ and it has been suggested that antibodies to one epitope may serve to inhibit binding to another neutralising epitope.^42^ It is intriguing to speculate that such inhibition could play a role in the context of natural infection in humans and could explain why the neutralisation is not inhibited by VD4_6-22_^D/E/F/G^ despite an anti-VD4_6-22_^D/E/F/G^ IgG response. Alternatively, only antibodies to adjacent non-neutralising epitope(s) were induced in these individuals.

Apart from the well-characterised species-specific VD4 epitope in MOMP, which is neutralising only in the B- and B-like-complex strains,^15^ neutralising epitopes have been identified in VD1, but these are restricted to the C-complex strains.^14^ The role of structural epitopes in MOMP has not been characterised in detail, but the superior effect of the native, purified MOMP compared to the recombinant protein in vaccine studies^33^ suggests that antibodies against structural epitopes in MOMP could also play a role. Other surface-exposed molecules could also be the neutralising target(s), and for Pmps, PorB, and OmcB this activity has been described.^43^, ^44^, ^45^, ^46^ In particular, PmpD has been described as an immunodominant antibody target after infection in humans,^47^ and the neutralising activity of vaccine induced antibodies against PmpD has been thoroughly characterised by Crane et al.^43^

For a future vaccine against genital CT infection, a systemic neutralising IgG response alone is likely not enough to protect against infection, although it could reduce the cervical bacterial burden.^8^ Antibody responses should be backed up by, and work in synergy with, a solid memory CD4 T cell response.^7^^,^^19^^,^^48^, ^49^, ^50^, ^51^, ^52^ A recent study with the CTH522/CAF®01 vaccine in mice and humans, demonstrated long-lasting immunity and protection one year after vaccination in mice, with an immune signature that translated to humans with respect to Th1/Th17 cytokine profile and antibody functionality.^30^ For the individuals infected with CT it would also be relevant to analyse the T cell responses to MOMP (epitope mapping, cytokine profile) and further extend the comparison of immune responses between infected and vaccinated individuals.

This study was limited in that the number of infected and vaccinated individuals studied was restricted (N = 10 and N = 14, respectively). Furthermore, in contrast to the described immunisation schedule for the vaccine recipients, the period between the time points for exposure and blood sampling is unknown for the individuals infected with CT but could play a role for the magnitude of the antibody response and perhaps the antibody signature. The response could also be influenced by repeated infections, in this study 29% self-reported a previous CT infection, but the actual number may be higher as CT infection is often asymptomatic.

The strength of this study is the direct comparison of the antibody responses induced after natural infection and vaccination, and the demonstration of important differences in antibody specificity and functionality. The results from our study may explain why infected individuals are not protected against reinfection despite a VD4 antibody response,^21^^,^^35^ and shows promise for the CTH522 vaccine as the response in vaccinated humans are similar to previous findings in animal models. Whether this vaccine approach of targeting specific epitopes rather than a broader response towards full length proteins represents a more general concept and could hold promise also for other pathogens will be the subject of future studies.

In summary, the CTH522/CAF®01-vaccinated participants had median UV-EB and NT_50_ titres approximately 4 times higher, respectively, compared to the individuals with natural genital infection. The ten infected MOMP-high-responders had comparable UV-EB, NT_50_ and VD4_6-22_^D/E/F/G^ titres, but lacked a consistent neutralising VD4 response.

## Contributors

IR, AWO, PA and FF did the conception and design of the study. AWO, HMC and RJS contributed with resources. IR, SK, ND, HBJ performed the experiments and the data analysis. IR, FF and AWO wrote the manuscript. All authors read and approved the final manuscript. IR and FF can verify the accuracy of the raw data for the study.

## Data sharing statement

The data generated for this study may be shared with other investigators upon written request to the corresponding author.

## Declaration of interests

The authors declare that the research was conducted in the absence of any commercial or financial relationships that could be construed as a potential conflict of interest. PA, AO, IR, and FF are co-inventors on a patent application relating to CT vaccines, and PA and IR are co-inventors on patents of the cationic adjuvant formulations (CAF). All rights have been assigned to Statens Serum Institut, a Danish not-for-profit governmental institute.

## Figures and Tables

**Supplemental Western blot Fig 1d_1:**
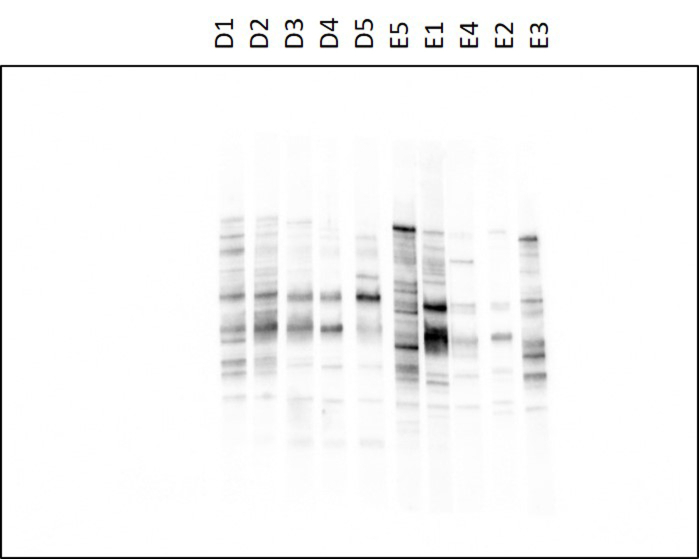
Uncropped Western blot image of left panel in Fig. 1d.

**Supplemental Western blot Fig 1d_2:**
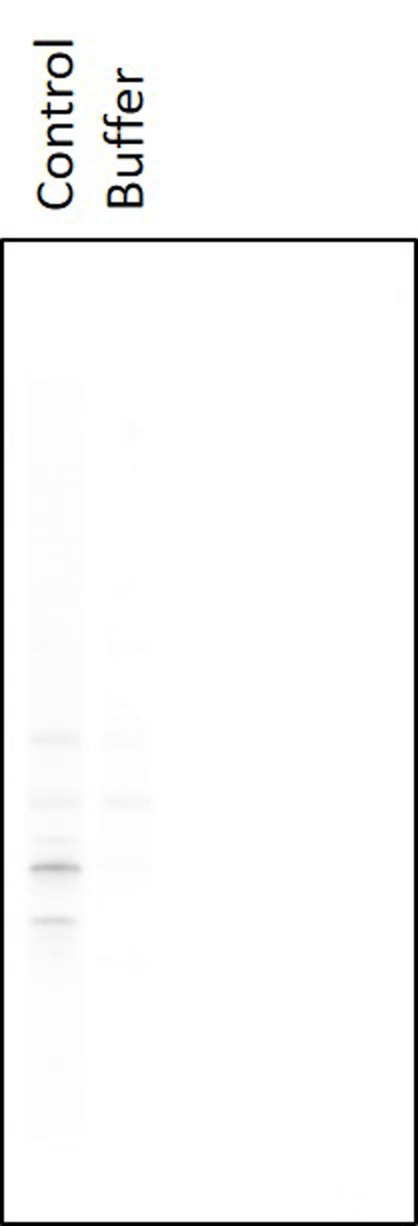
Uncropped Western blot image of right panel in Fig. 1d.
