# Supplementary material for: Human antibody signatures towards the Chlamydia trachomatis major outer membrane protein after natural infection and vaccination
Source: eBioMedicine. 2024 May 13;104:105140. doi: 10.1016/j.ebiom.2024.105140 (PMC11108849; doi:10.1016/j.ebiom.2024.105140)
Supplement: Supplemental Fig. S1–S4 and Table S1 [file mmc1.pdf]

Supplementary Information for 'Human Antibody Signatures towards the *Chlamydia trachomatis* Major Outer Membrane Protein after Natural Infection and Vaccination' by Rosenkrands et al

Supplemental table 1. Characteristics of participant infected with CT at enrolment (n=80)

	Male		Female		All	
Characteristic	N	(%)	N	(%)	N	(%)
	36	45	44	55	80	100
Age (years)						
16-25	11	31	27	61	38	48
>25	25	69	17	39	42	53
Previous infection reported						
No	26	72	31	70	57	71
Yes	10	28	13	30	23	29
Symptoms before diagnosis reported						
No	16	44	30	68	46	58
Yes	20	56	14	32	34	43
Genotyping of strain						
D	9	25	10	23	19	24
E	11	31	16	36	27	34
F	7	19	9	20	16	20
G	5	14	1	2	6	8
H	0	0	3	7	3	4
I	1	3	0	0	1	1
J	1	3	1	2	2	3
K	2	6	4	9	6	8

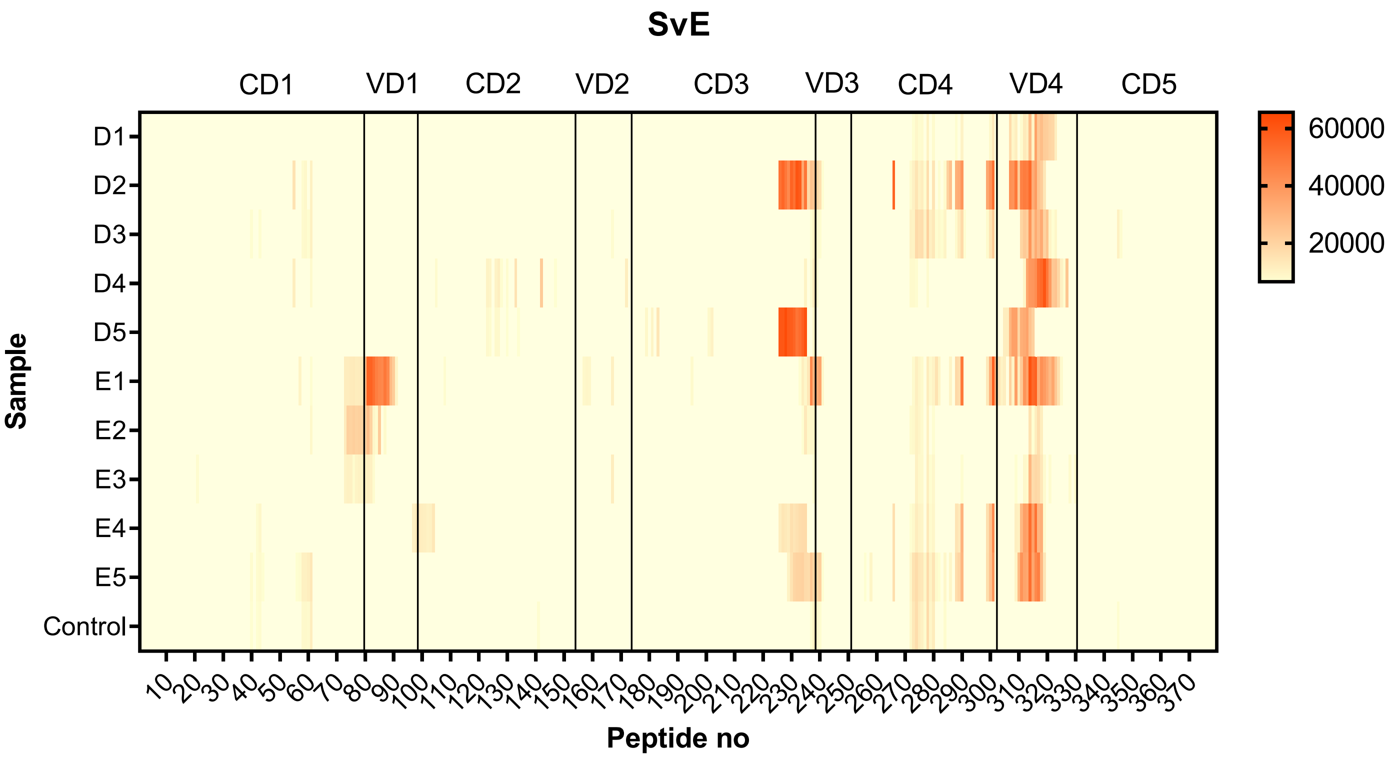

Supplemental Fig. 1. IgG responses to MOMP SvE 15mer overlapping peptides recognised by individuals infected with CT named according to the genotype of the infecting strain (D1-D5 and E1-E5). A heat map is shown for a negative control pool and 10 selected individuals infected with CT with a high response to SvD MOMP.

A

SvD MOMP

MKKLLKSVLVFAALSSASSLQALPVGNPAEPSLMIDGILWEGFGGDPCDPCATWCDAISMRVGYYGDFVF
DRVLKTDVNKEFQMGAKPTTDTGNSAAPSTLTARENPAYGRHMQDAEMFTNAACMALNIWDRFDVFCTLG
ATSGYLKGNSASFNLVGLFGDNENQKTVKAESVPNMSFDQSV**VELYTDTTFAW**SVGARAALWECGCATLG
ASFQYAQSKPKVEELNVLCNAAEFTINKPKGYVGKEFPLDLTAGTDAATGTKDASIDYHEWQASLALSYR
LNMFTPYIGVKWSRASFDADTIRIAQPKSATAIFDTTTLNPTIAGAGDVKTGAEGQLGDTMQIVSLQLNK
MKSRKSCGIAVGTTIVDADKYAVTVETRLIDERAAHVNAQFRF

B

SvE MOMP

MKKLLKSVLVFAALSSASSLQALPVGNPAEPSLMIDGILWEGFGGDPCDPCTTWCDAISMRMGYYGDFVF
DRVLKTDVNKEFQMGDKPT**STTGN**ATAPTTLTARENPAYGRHMQDAEMFTNAACMALNIWDRFDVFCTLG
ASSGYLKGNSASFNLVGLFGDNENQSTVKTNSVPNMSLDQSVVELYTDTAFSWSVGARAALWECGCATLG
ASFQYAQSKPKVEELNVLCNAAEFTINKPKGYVGQEFPLALIAGTDAATGTKDASIDYHEWQASLALSYR
LNMFTPYIGVKWSRASFDADTIRIAQPKSATAIFDTTTLNPTIAGAGDVKASAEGQLGDTMQIVSLQLNK
MKSRKSCGIAVGTTIVDADKYAVTVETRLIDERAAHVNAQFRF

VD1
VD2
VD3
VD4

Supplemental Fig. 2. The antigenic regions identified by peptide array in individuals infected with CT in MOMP SvD (A) and MOMP SvE (B) are emphasised in yellow. Antigenic regions specific for one serovar are shown in bold face. Variable domain regions are underlined in the colors shown below, the signal sequence is shown as grey text.

CTH522

MDAISMRVGYYGDFVFDRVLKTDVNKEFQMGAKPTTDTGNSAAPSTLTARENPAYGRHMQDAEMFTNAASMALN
IWDRFDVFSTLGATSGYLKGNSASFNLVGLFGDNENQKTVKAESVPNMSFDQSVVELYTDTTFAWSVGARAALW
ESGSATLGASFQYAQSKPKVEELNVLSNAAEFTINKPKGYVGKEFPLDLTAGTDAATGTKDASIDYHEWQASLA
LSYRLNMFTPYIGVKWSRASFDADTIRIAQPKSATAIFDTTTLNPTIAGAGDVKTGAEGQLGDTMQIVSLQLNN
MFTPYIGVKWSRASFDADTIRIAQPKSATAIFDTTTLNPTIAGAGDVKASAEGQLGDTMQIVSLQLNNMFTPYI
GVKWSRASFDSDTIRIAQPRLVTPVVDITTLNPTIAGSGSVAGANTEGQISDTMQIVSLQLNNMFTPYIGVKWS
RASFDSNTIRIAQPKLAKPVVDITTLNPTIAGSGSVVAANSEGQISDTMQIVSLQLN

VD1
VD2
VD3
VD4

Supplemental Fig 3. The antigenic regions identified in CTH522 by peptide array in vaccinated individuals are emphasised in yellow. Variable domain regions are underlined in the colors shown below.

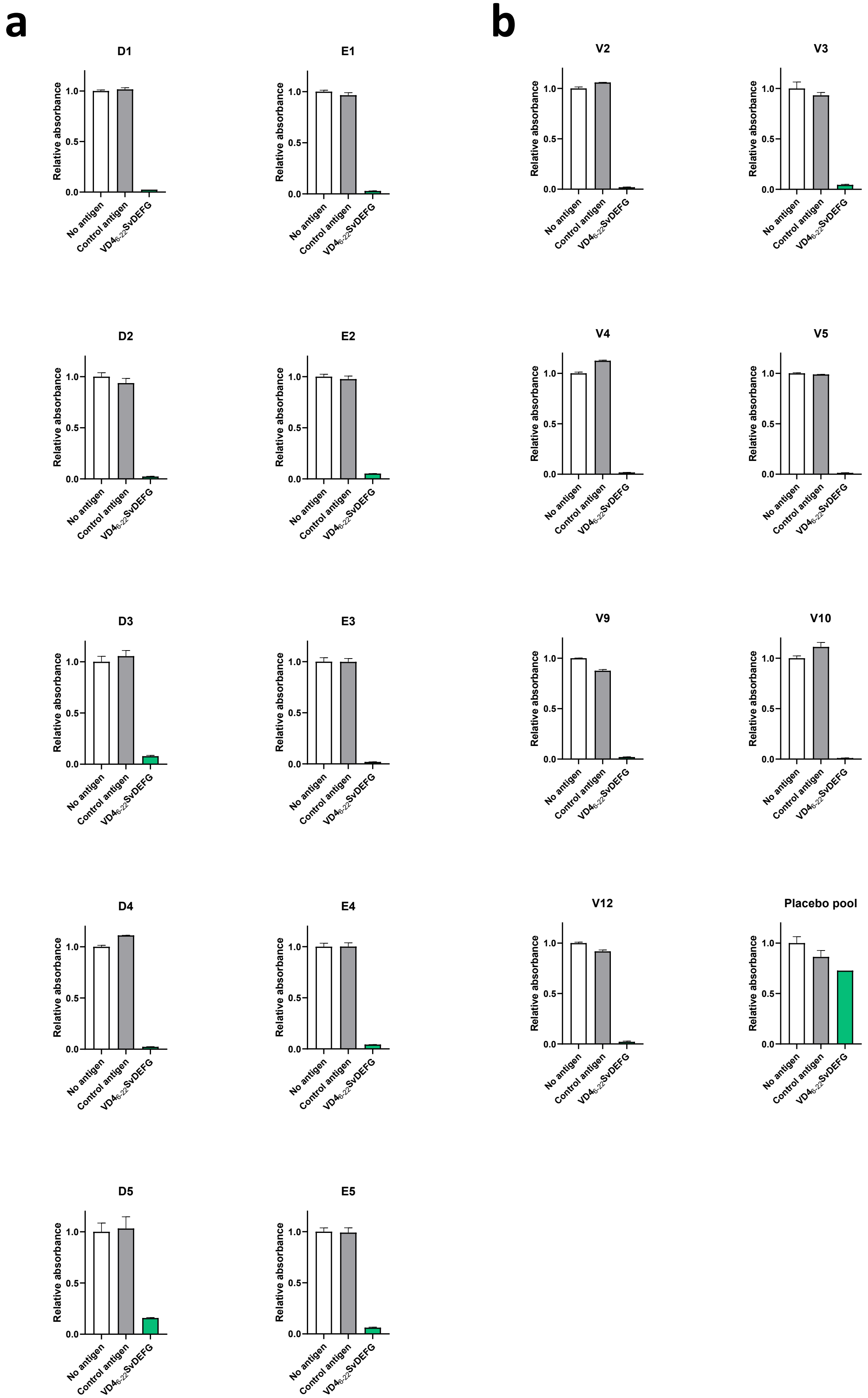

Supplemental Fig. 4. Blocking of VD4$_{6\text{-}22}^{D/E/F/G}$ ELISA response in individuals infected with CT (a) and vaccinated (b). ELISA plates were coated with VD4$_{6\text{-}22}^{D/E/F/G}$ and samples were diluted corresponding to 80-90% neutralisation against SvD and pre-incubated with 160 µg/ml of control antigen, VD4$_{6\text{-}22}^{D/E/F/G}$ or no antigen before the standard ELISA. For each antigen, absorbance relative to that of the No antigen is shown. Results are presented as mean plus standard deviation.
